# Musculoskeletal Disorders in Chronic Obstructive Pulmonary Disease

**DOI:** 10.1155/2014/965764

**Published:** 2014-03-25

**Authors:** Nele Cielen, Karen Maes, Ghislaine Gayan-Ramirez

**Affiliations:** Respiratory Muscle Research Unit, Laboratory of Pneumology and Respiratory Division, Department of Clinical and Experimental Medicine, Katholieke Universiteit Leuven, Leuven, Belgium

## Abstract

Chronic obstructive pulmonary disease (COPD) is a lung disease characterized by airway obstruction and inflammation but also accompanied by several extrapulmonary consequences, such as skeletal muscle weakness and osteoporosis. Skeletal muscle weakness is of major concern, since it leads to poor functional capacity, impaired health status, increased healthcare utilization, and even mortality, independently of lung function. Osteoporosis leads to fractures and is associated with increased mortality, functional decline, loss of quality of life, and need for institutionalization. Therefore, the presence of the combination of these comorbidities will have a negative impact on daily life in patients with COPD. In this review, we will focus on these two comorbidities, their prevalence in COPD, combined risk factors, and pathogenesis. We will try to prove the clustering of these comorbidities and discuss possible preventive or therapeutic strategies.

## 1. Introduction

The Global Initiative for Chronic Obstructive Lung Disease (GOLD) defines chronic obstructive pulmonary disease (COPD) as “a preventable and treatable disease, characterized by a persistent airflow limitation that is progressive and not fully reversible and associated with an abnormal inflammatory response of the lungs to noxious gases or particles. Exacerbations and comorbidities contribute to the overall severity in individual patients” [1]. Currently, COPD is the fourth leading cause of death in the world and will rise to the third leading cause of death by 2030 [2].

COPD is spirometrically diagnosed by the presence of a postbronchodilator FEV_1_/FVC < 0.70 and is assessed for its severity according to FEV1 level: mild COPD (FEV_1_ ≥ 0.80 predicted), moderate COPD (0.50 ≤ FEV_1_ ≤ 0.80 predicted), severe COPD (0.30 ≤ FEV_1_ ≤ 0.50 predicted), and very severe COPD (FEV_1_ < 0.30 predicted) [1]. In 2013, a new classification method has been developed which combines spirometric classification with symptomatic assessment (through the modified British Medical Research Council (mMRC) questionnaire or COPD Assessment Test (CAT)) and/or with exacerbation risk [1]. All the literature discussed in this review is based on the old classification system.

Although COPD is primarily a lung disease, it is associated with comorbidities such as cardiovascular disorders, metabolic diseases (diabetes mellitus, metabolic syndrome, and obesity), chronic kidney disease, sleep apnoea, anemia, depression, lung cancer, weight loss, skeletal muscle weakness, and osteoporosis. These comorbidities contribute to a reduced health status, increased healthcare utilization and hospital admission, and mortality [3].

In this review, we will focus on skeletal muscle weakness and osteoporosis in patients with COPD. Risk factors and pathogenesis contributing to both comorbidities, as well as therapeutic strategies, will be discussed.

## 2. Skeletal Muscle Weakness and Osteoporosis in COPD

### 2.1. Definition and Prevalence

Skeletal muscle function is described by muscle strength (the ability to generate force production), muscle endurance (the ability to sustain a given contraction over time), and muscle fatigue (a physiological sense defined as the failure of force generation resulting from activity under load which is reversible by rest). In COPD, skeletal muscle weakness is characterized by reduced muscle strength, reduced muscle endurance, and the presence of muscle fatigue [4]. The estimated overall prevalence of skeletal muscle weakness in patients with COPD was shown to be 32% [5]. In addition, a trend towards higher prevalence of skeletal muscle weakness with disease severity (GOLD stages) has been reported [5]. Skeletal muscle weakness was shown to contribute to decreased functional capacity, poor quality of life, increased healthcare utilization, and even mortality [3], independently of lung function [6].

The World Health Organization defines osteoporosis as a systemic disease, characterized by a low bone mineral density and/or microarchitectural deterioration of bone tissue, leading to increased bone fragility and fracture risk [7]. The prevalence in patients with COPD varies between 9 and 69%, depending on the population studied, diagnostic methods used, and the definition used to define osteoporosis [8]. Prevalence increases with the severity of COPD [9–11]. Two types of fractures are related to osteoporosis. Peripheral fractures or hip fractures impair mobility, while vertebral fractures lead to back pain and indirectly decline pulmonary function due to decreased rib mobility [12, 13]. Fractures are a substantial cause of morbidity and lead to functional decline, loss of quality of life, need for institutionalization, and mortality [14]. Since osteoporosis is highly common in patients with COPD [15], the impact of these events may be even worse.

### 2.2. Clinical Evidence for Skeletal Muscle Weakness and Osteoporosis in COPD

**Figure 1:**
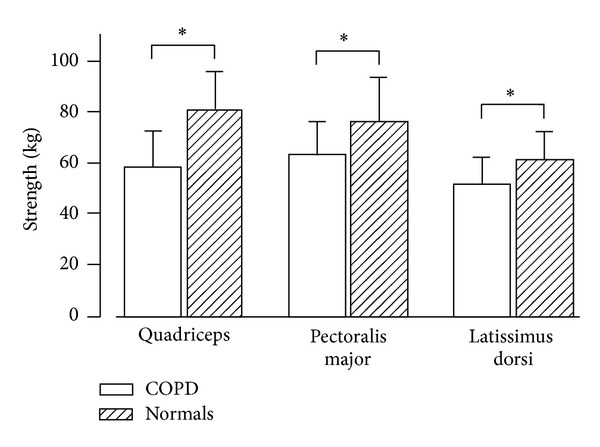
Reduced muscle strength of the quadriceps, pectoralis major, and latissimus dorsi obtained in normal subjects and patients with COPD. All three types of muscles show decreased muscle strength in patients with COPD. *P < 0.005 Reprinted with permission of the American Thoracic Society. Copyright © 2014 American Thoracic Society, official journal of the American Thoracic Society [17].

**Figure 2:**
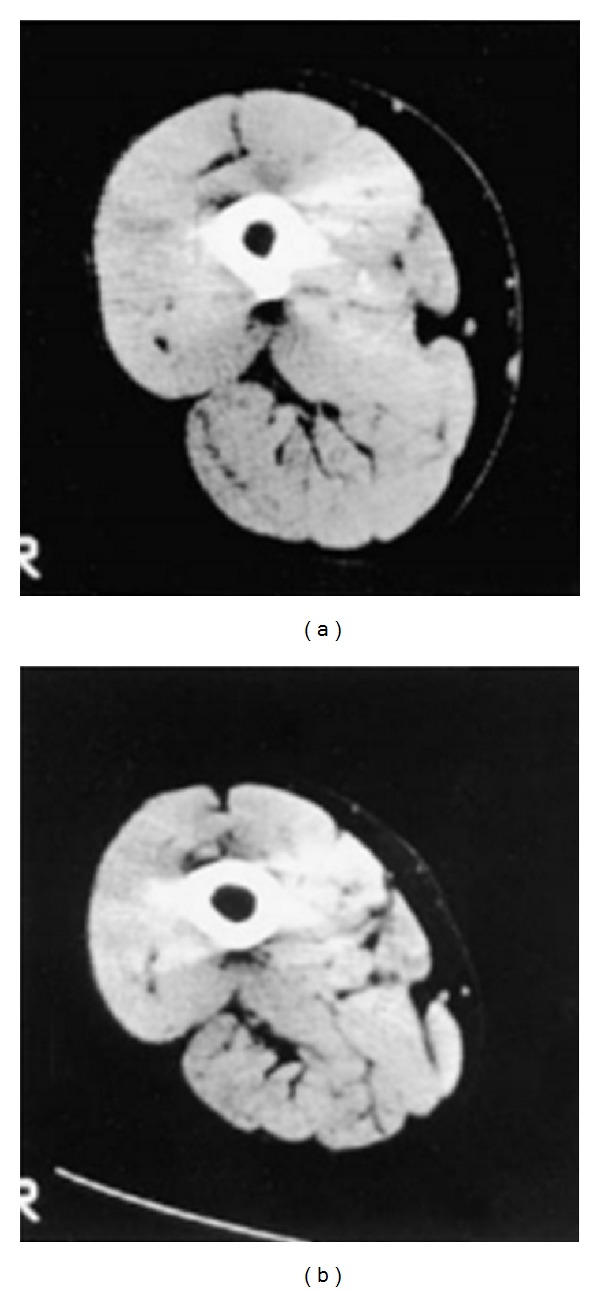
Cross-sectional area as well as muscle force of thigh muscle was reduced in patients with COPD (b) compared to normal subject (a). Reprinted with permission of the American Thoracic Society. Copyright © 2014 American Thoracic Society, official journal of the American Thoracic Society [17].

**Figure 3:**
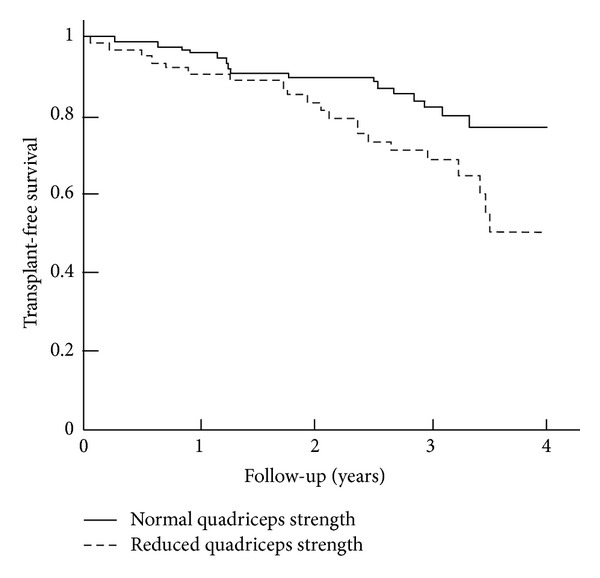
Survival of patients with COPD with normal and reduced quadriceps strength. The curves are significantly different *P* = 0.017 [24].

Skeletal muscle weakness is reflected by reduced muscle strength (Figure 1) and endurance and increased muscle fatigability [16]. Muscle weakness is mainly observed in the lower limb muscle of patients with COPD [17]. Indeed, quadriceps muscle weakness is a common feature in patients within all stages of COPD [5] in both men and women [18]. Lower limb muscle weakness is found to be more severe in patients with cachexia [19] and worsens during acute exacerbations [20, 21]. The structure and function of the upper limb muscles are found to be relatively preserved [22] (Figure 1), even when patients are in a cachectic state [19], but not during acute exacerbations where strength of upper limb muscles was found to be reduced [21]. Preservation of upper limb muscle in stable COPD is most probably due the fact that those muscles are involved in daily activities [23]. In lower limb muscles, several adaptations develop with COPD; these include muscle fiber type shift from type I towards type IIx muscle fibers resulting in reduced oxidative and increased glycolytic capacity, fiber atrophy, loss of muscle mass (Figure 2), and decreased capillary density [4]. Importantly, reduced quadriceps strength is found to be a useful predictor for mortality in patients with COPD (Figure 3) [24].

**Figure 4:**
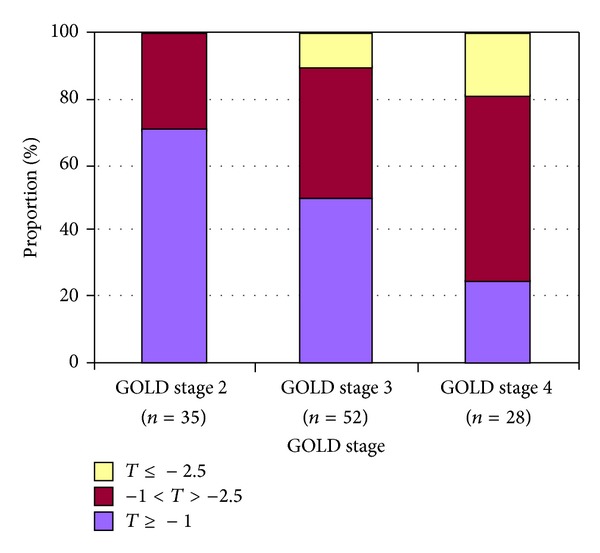
Bone mineral density in different GOLD stages. The *T*-score is the difference between the patient's results and the mean results obtained in a young population, expressed in units of standard deviation. Osteoporosis is the condition with a *T*-score below −2.5; osteopenia is the condition of a *T*-score between −1 and −2.5. The prevalence of low bone mineral density increases with higher GOLD stage [25].

Osteoporosis is common in both male and female patients with COPD [15]. Moreover, the risk of developing osteoporosis was found to be associated with airflow obstruction [26]. COPD was also shown to be a significant independent predictor for reduced bone mineral density (Figure 4) and increased fracture risk [27], and both are associated with the severity of the disease [11, 28].

There is evidence of a possible direct mechanistic link between COPD and osteoporosis. This is highlighted in studies using CT-scan, indicating a relationship between pulmonary emphysema and reduced bone mineral density [29, 30], independently of airflow obstruction and other osteoporosis risk factors [31].

### 2.3. Risk Factors for Skeletal Muscle Weakness and Osteoporosis

**Figure 5:**
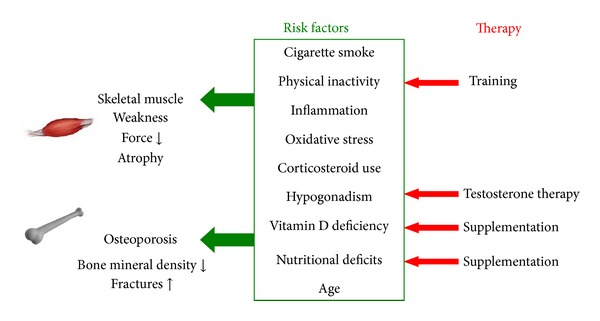
Several risk factors, such as cigarette smoke, physical inactivity, inflammation and oxidative stress, corticosteroid use, hormonal disturbances, nutritional deficits, and age, lead to the development of skeletal muscle weakness and osteoporosis in healthy individuals, as well as patients with COPD. There are several therapy modalities that can be used to treat or inverse the consequences of these risk factors so that the risk of developing skeletal muscle weakness and osteoporosis can be reduced in patients with COPD.

There are several risk factors in COPD that may contribute to both skeletal muscle weakness and osteoporosis. These include cigarette smoking, physical inactivity, systemic and local inflammation, oxidative stress, corticosteroid use, hormonal disturbances, and age (Figure 5).

#### 2.3.1. Cigarette Smoke

The most important risk factor for the development of COPD is cigarette smoke. Twenty percent of the world population smokes cigarettes [32] (http://www.tobaccoatlas.org/) and 90% of patients with COPD either have a history of smoking or still smoke.

Cigarette smoke is an important contributor to skeletal muscle weakness and it has been shown to exert negative effects on bone. Cigarette smoke was shown to be related to decreased skeletal muscle strength and physical performance in healthy adults [33, 34]. Compared to controls matched for age and physical activity, healthy young smokers showed a reduced fatigue resistance of the quadriceps muscle [35]. In healthy smokers and patients with COPD, cigarette smoke was shown to induce muscle atrophy, reduce muscle protein synthesis, induce oxidative modifications on muscle proteins [36], and increase the expression of genes involved in muscle catabolism and associated with inhibition of muscle growth [37]. Also in studies with animals chronically exposed to cigarette smoke, muscle atrophy developed [38].

In healthy smokers, cigarette smoke was found to compromise bone strength [39] and to be associated with reduced bone mineral density [40], increased risk of fractures, and delayed fracture healing at all skeletal sites. The association was present in current as well as former smokers [41] and the risk for osteoporosis was stronger with higher cigarette consumption. The effects of cigarette smoke on bone mineral density and fracture risk were observed in both men and women but were reported to be more deleterious in men [40]. In addition, this effect was found to be independent of age [42]. The effect of cigarette smoke on bone mineral density and bone turnover was confirmed by animal studies [43, 44].

#### 2.3.2. Physical Inactivity

Daily physical activity is significantly reduced in patients with COPD, compared to healthy age-matched controls. Indeed, patients with COPD spent more time sitting and lying and less time walking (<50 minutes daily versus 81 minutes) and standing [45]. Daily physical activity is already reduced in patients with GOLD stage I, and this reduction further worsens with disease severity, with patients with COPD with the GOLD stage IV being very inactive [46–48]. Importantly, patients with COPD with very low physical activity had a higher risk for hospital admission and mortality [49, 50]. Finally, these patients are very inactive during hospitalization for an acute exacerbation (daily walking time becomes <10 minutes) and remained inactive even one month after discharge [51], thereby increasing the risk for readmission [52].

Physical inactivity was found to be crucial in the development of skeletal muscle weakness in patients with COPD. It is believed to result in quadriceps weakness due to mechanical unloading of the muscle and due to muscle wasting [17, 53] and it was shown to be associated with impaired muscle endurance [54]. The reduced lower leg activity observed in patients with COPD was shown to be related to total daily activity [55]. It may contribute to impaired physical balance [56] and to increased risk of falling [57] in patients with COPD. Decreased physical activity has been suggested as a possible link between low body composition and osteoporosis or low bone mineral density in patients with COPD. Alternatively, decreased bone formation due to a reduction in mechanical loading on the bones may play a role too [8]. In elderly healthy individuals, it was shown that physical inactivity was related to a high rate of bone loss and with the risk of fractures [58] but there are no direct studies yet in patients with COPD.

#### 2.3.3. Systemic and Local Inflammation

Systemic inflammation has been reported in patients with severe COPD with muscle wasting. It is characterized by increased serum levels of tumor necrosis factor (TNF)-*α*, its receptors [59], interleukin (IL)-1*β* [60], IL-6, IL-8, IL-18 [61], and acute phase reactants [62]. Interestingly, in patients with COPD who are hospitalized for acute exacerbations, increased serum levels of IL-8 were found to be negatively associated with quadriceps weakness, while IL-6 levels remained unaltered and TNF*α* was not detectable [21].

The presence of local inflammation in the muscle of patients with COPD is still controversial. Some studies reported an upregulation of TNF*α* [63, 64] in the quadriceps muscle, while other studies did not [20, 61, 65, 66], even during acute exacerbations [20]. Also, IL-6 and IL-8 expression in the muscle remained unaltered during acute exacerbations [20]. In patients with stable COPD, IL-18 was shown to be upregulated in the vastus lateralis muscle [61]. But cytokine profile in the quadriceps of weight stable patients with severe COPD did not show any presence of a proinflammatory environment [65]. Along the same line, a microarray analysis did not reveal any upregulation of inflammatory markers in the muscle of patients with COPD during exacerbations [67].

#### 2.3.4. Oxidative Stress

Oxidative stress occurs when the balance between oxidant production (reactive oxygen species) and antioxidant capacity in the cell is disturbed, causing damage of lipids, proteins, and DNA [68]. The most important triggers for the development of oxidative stress in patients with COPD are cigarette smoke and systemic inflammation [69].

Several markers of oxidative stress were shown to be upregulated in the muscle and plasma of patients with stable COPD [65, 70, 71], and these were further enhanced in plasma during exacerbations [72, 73]. On the other hand, the antioxidant capacity was found to be increased in the muscles of patients with COPD, probably because the defense system was triggered by exposure to reactive oxygen species [74]. Importantly, oxidative stress was found to be associated with decreased quadriceps muscle strength [36, 75] and was shown to cause increased bone resorption during severe COPD exacerbations [76]. But whether oxidative stress directly affects bone mineral density and osteoporotic fractures in these patients is not yet known.

#### 2.3.5. Corticosteroid Use

Corticosteroids are frequently used in patients with COPD to reduce pulmonary symptoms and to treat exacerbations [77]. Steroid-induced myopathy and steroid-induced osteoporosis represent the well-known side effects of corticosteroid treatment.

Two types of steroid-induced myopathy have been described: acute or chronic. Acute steroid myopathy is rare in COPD. It represents a complication of treatment with systemic corticosteroids [78], leading to proximal as well as distal muscle weakness, occurring after 5–7 days of high dose (hydrocortisone 1–4 g/day or dexamethasone 40 mg/day) intravenous treatment. After treatment cessation, the recovery is prolonged up to 6 months [79, 80]. Chronic steroid myopathy occurs after long-term treatment with low doses of oral corticosteroids and results in proximal muscle weakness and generalized muscle fiber atrophy [81, 82]. This myopathy is more frequently observed with fluorinated corticosteroids. Recovery of this type of myopathy may be spread over weeks to months. On follow-up, survival of patients with steroid-induced myopathy was reduced in comparison to COPD with a similar degree of airflow obstruction [82]. In patients with COPD receiving daily low doses of oral corticosteroids, a negative relationship was found between corticosteroid use and skeletal muscle strength [81]. Oral corticosteroid treatment was found to contribute to loss of fat-free mass [83], which is an independent predictor of mortality in patients with COPD [84].

Oral corticosteroids were found to be inversely correlated with bone mineral density, and the daily dose was correlated with the risk of osteoporotic fractures [85]. The risk of fractures was found to increase within 3 to 6 months after the start of the therapy and decreased after therapy cessation [85]. Bone mineral density was found to be decreased in patients receiving multiple courses of oral or intravenous glucocorticosteroids [86]. In patients with COPD, a long-term treatment with inhaled corticosteroids had no effect on fracture risk [87] at conventional doses [88]. One year of inhaled corticosteroid treatment was shown to exert no effects on bone mineral density [89] while a treatment of 3 years with inhaled triamcinolone was found to reduce bone mineral density [90].

#### 2.3.6. Hormone Disturbances

*(1) Hypogonadism*. The prevalence of hypogonadism (total testosterone levels <280–300 ng/dL and free testosterone level <5 pg/mL [91, 92]) in men with COPD varies between 22 and 69% [93] and is unknown in women. The most important risk factors to develop hypogonadism are smoking, hypoxia, systemic inflammation, and corticosteroid treatment, risk factors that are also related to COPD [93].

In male individuals without COPD, hypogonadism was shown to be associated with the loss of muscle mass and strength, increased prevalence of osteoporosis, and accelerated bone loss [42, 94]. In patients with COPD, low levels of testosterone are associated with reduced quadriceps strength, but preserved exercise capacity, when compared to healthy age-matched controls [92]. Since low testosterone levels are associated with osteoporosis, and osteoporosis is common in COPD, a potential link might be suggested although this has not yet been investigated.

*(2) Vitamin D Deficiency*. Vitamin D is essential for bone and muscle health while regulating calcium, phosphate, and bone homeostasis [95]. It was shown to play an important role in the growth of skeletal muscles, muscle contractility, and myogenesis [96] as well as in the development of the growth plate, mineralized bone, and osteoclastogenesis [97].

In humans, vitamin D deficiency, defined as serum levels below 20 ng/mL (50 nmol/L) was found to be associated with poor muscle strength and performance [98] and decreased physical activity [99]. Due to an imbalance in calcium and phosphate homeostasis, vitamin D deficiency is also known to be a risk factor for severe osteoporotic fractures [100]. In addition, an association between a polymorphism of the vitamin D receptor and bone mineral density has been highlighted [101].

The prevalence of vitamin D deficiency in patients with severe and very severe COPD is, respectively, 60% and 77% [102]. Vitamin D deficiency was found to be correlated with disease severity [102, 103] but not with acute exacerbations and mortality [104, 105]. Several reasons could account for vitamin D deficiency in patients with COPD, including a poor diet, reduced capacity of the aging skin to synthesize vitamin D, absence of outdoor activity and sun exposure, increased catabolism by glucocorticosteroids, impaired activation due to renal dysfunction, and lower storage capacity in muscles or fat, due to wasting [106].

In patients with COPD, a relationship was found between variants in the vitamin D receptor gene and skeletal muscle strength [107] but, although an association was observed between vitamin D levels and muscle strength in control patients, this association was not present in patients with COPD [108, 109]. This observation might indicate that some patients with COPD may be resistant to the actions of vitamin D, which was corroborated by elevated levels of PTH in these patients [108]. Plasma concentration of vitamin D was found to be positively correlated with bone mineral density and functional exercise capacity in patients with COPD [109], and with an increased risk of osteoporosis in these patients [110].

#### 2.3.7. Nutritional Deficits

In patients with stable COPD, the prevalence of undernutrition was estimated between 20% and 27% [111, 112], and up to 35% in patients admitted to a pulmonary rehabilitation program [113]. It was found to be more prevalent in female patients with moderate to severe COPD [112].

Undernutrition in patients with COPD can be caused by inadequate dietary intake, which was found to be worse during an acute exacerbation [114], and enhanced energy expenditure. Other causes are a loss of appetite, anorexia, and the effects of humoral factors, such as inflammatory cytokines, adipokines, and hormones [113].

Undernourished patients with stable COPD were found to have lower skeletal muscle strength [111]. Importantly, in patients with COPD who are hospitalized for acute exacerbations, malnutrition was highly prevalent [115] and was found to increase the risk for having new exacerbations [116] and the risk for rehospitalization and mortality [117].

When marked undernutrition occurs, the distortion of the energy balance was found to cause cachexia, the involuntary loss of over 5% of bodyweight [118]. In patients with COPD, loss of body weight correlated with disease severity [83, 119, 120] and skeletal muscle weakness [121] independently of airflow obstruction [6]. Decreased body weight has been identified as a poor prognostic factor in patients with COPD [120, 122]. Similarly, low fat-free mass and low body mass index were found to be related with bone mineral density loss [25, 123] and increased risk for developing osteoporosis [9, 124, 125] in patients with COPD.

#### 2.3.8. Age

In healthy subjects, age is known to negatively impact muscle strength and contraction speed [126]. It also induces a shift from muscle type II to type I fibers and atrophy of type II fibers [127, 128]. As a consequence, limb muscles of elderly are smaller and contain more fat and connective tissue [126]. With age, the incidence of fractures is also found to increase while bone mineral density decreases [129].

In patients with COPD, increasing age was found to reduce quadriceps strength [130] and this loss of strength was more pronounced than that of age-matched healthy individuals [131]. Important to emphasize is that the muscle fiber shift in patients with COPD (from type I to type II fibers) is opposite to the shift induced by age. Increasing age was also found to be associated with an increased risk for osteoporosis in patients with COPD [124].

### 2.4. Clustering of Skeletal Muscle Weakness and Osteoporosis in a Subgroup of Patients with COPD

COPD is commonly associated with one or more comorbidities [132–134]. Several studies highlighted clustering of some of these comorbidities in subgroup of patients with COPD [3, 134]. As such, skeletal muscle weakness, osteoporosis, and cachexia were described as a cluster in a group of patients with COPD [3]. Similarly, in a study where the frequency and clustering of 13 relevant comorbidities of COPD have been investigated [134], underweighted patients with COPD were found to have a high prevalence of osteoporosis (57%) and muscle wasting (93%). Fifty percent of the patients with COPD with osteoporosis were found to have muscle wasting, while 55% of the patients with muscle wasting also suffered from osteoporosis [134].

Cachexia is a complex metabolic syndrome associated with an underlying illness and characterized by loss of muscle with or without loss of fat mass, often combined with signs of systemic inflammation, and anorexia [118]. Cachexia occurs in 20% to 40% of the patients with COPD [135].

Cachexia was found to be associated with poor functional capacity, reduced quality of life [136], and increased risk for morbidity and mortality in patients with COPD [84, 137–141]. It is independently correlated with osteopenia and osteoporosis in elderly patients [124]. The risk factors for cachexia are multifactorial and include a disturbed energy balance, oxidative stress, systemic inflammation, hypogonadism, and corticosteroid treatment [142]. Because of the similarity in risk factors, as described before, the possibility of the clustering of skeletal muscle weakness, osteoporosis, and cachexia in a subgroup of patients with COPD is emphasized. It is not clear yet whether cachexia is associated with changes in protein synthesis, but several proteolytic markers (Atrogin-1, MuRF1) were found to be increased in muscles of cachectic patients with COPD together with a decreased expression of myogenic differentiation factors [135]. Weight loss is found to cause bone loss [143], but the exact mechanism is unknown. But cachexia is associated with muscle loss, causing decreased mechanical loading on bones, which is a risk factor for osteoporosis. On the other hand, cachexia is also associated with systemic inflammation, which is also found to be a risk factor for osteoporosis.

Interestingly, there is also evidence for associations between muscle strength and bone mineral density in other diseases, such as cystic fibrosis [144], Crohn's disease [145], acute lymphoblastic leukemia [146], and osteoporosis itself [147–150].

### 2.5. Pathogenesis

Cigarette smoke may affect skeletal muscle and bone through its toxic components, while inducing oxidative stress and systemic inflammation [37]. Nicotine is the major toxic component of cigarette smoke and may interact with the nicotine acetylcholine receptor in many cells. At concentrations of nicotine equivalent to levels found in blood of heavy smokers, nicotine reduced cell proliferation and downregulated genes associated with osteogenesis [151], thereby impairing bone strength and bone mass [152]. Nicotine is also found to induce insulin resistance and to decrease insulin release by pancreatic beta cells. Insulin modulates protein synthesis and degradation in the muscle, and therefore, insulin resistance is found to promote catabolism of the skeletal muscle [37]. Chronic exposure to nicotine also decreases the total Na-K ATPase activity, thereby depolarizing the membrane in the skeletal muscle [153].

Cigarette smoke also contains reactive oxygen and nitrogen-free radicals, causing imbalance between oxidants and antioxidants and leading to oxidative stress. The latter is known to induce modification of proteins, lipids, and DNA. In patients with COPD, levels of protein carbonylation and nitration, lipid peroxidation, and protein oxidation are found to be elevated in blood and limb muscles [154]. Oxidative stress is found to be involved in reduced quadriceps endurance [155] and protein oxidation to contribute to muscle loss and dysfunction [36]. Oxidative stress in skeletal muscle may lead to increased muscle proteolysis, through the upregulation of E3 ligases (MAFbx/Atrogin-1 and MuRF-1), thereby activating the ubiquitin proteasome system [37]. These ligases are both increased in skeletal muscle of patients with COPD [135]. On the other hand, the muscular antioxidant status is found to be altered in patients with COPD as a compensation for increased ROS formation [74]. In patients with COPD, oxidative stress also affects the protease/antiprotease balance, inducing inactivation of antiproteases, and activation of metalloproteinases [156]. Matrix metalloproteinases (MMP) have regulatory functions in bone turnover. MMP9 levels are increased in patients with COPD [76] and are related to osteoporosis, through the activation of osteoclasts [157].

Cigarette smoke also induces systemic inflammation through activation of circulating inflammatory cells and release of inflammatory mediators into the circulation [158]. In patients with stable COPD, enhanced IL-6 and TNF*α* levels are associated with reduced quadriceps strength and exercise capacity [159, 160] as well as muscle wasting [161–164]. These increased IL-6 and TNF*α* levels are also stimulating bone resorption [165] and inhibiting bone formation, thereby lowering bone mineral density [166, 167]. Increased CRP serum levels are also associated with reduced quadriceps strength and exercise capacity in patients with stable COPD. CRP also plays an important role in osteoporosis [168]. IL-1 was found to play an important role in osteoclast action by increasing the production of the macrophage colony stimulating factor (a regulator of osteoclastogenesis) and by inhibiting osteoclast apoptosis [169].

Cigarette smoking is also known to be associated with low levels of physical activity [170]. In fact, physical inactivity is common in patients with COPD and starts early in the development of the disease. It might aggravate skeletal muscle weakness and osteoporosis while causing mechanical unloading of muscle and bone. Reduced mechanical loading on bone is found to inhibit osteoblast-mediated bone formation and to accelerate osteoclast-mediated bone resorption, causing disuse osteoporosis [171]. Physical inactivity is also found to lead to increased oxidative stress in the skeletal muscle [172].

Since many patients with COPD have reduced physical activity levels, they will spend less time on outdoor activities, whereby the amount of sun exposure will decline. This could lead to vitamin D deficiency, which is also highly prevalent in patients with COPD.

All these effects combined with the presence of other risk factors worsen skeletal muscle function and bone health in patients with COPD, causing muscle weakness and osteoporosis.

### 2.6. Possible Preventive and Therapeutic Strategies

#### 2.6.1. Pharmacological Interventions

*Testosterone Replacement Therapy*. Testosterone therapy (Figure 5) has been shown to increase muscle protein synthesis in elderly men [173], to increase muscle mass [174], and muscle strength in healthy and hypogonadal men [175, 176]. In addition, testosterone therapy was able to enhance bone mineral density in healthy and hypogonadal men [177, 178] by suppressing bone resorption [179, 180] and bone remodeling [181] due to the inhibition of IL-6 expression [182].

In patients with COPD, testosterone therapy (80–100 mg/week, 25–50 mg/2 weeks, or 250 mg/4 weeks) was shown to improve peak muscle strength [183, 184] and to increase body weight and fat-free mass [185, 186]. This increased muscle mass and strength was not necessarily translated into improved functional capacity. There are no studies dealing with the effect of testosterone therapy on osteoporosis, quality of life, or survival in patients with COPD.

Testosterone therapy is found to be accompanied by several deleterious side effects such as increased hemoglobin and hematocrit levels and a small decrease in high-density lipoprotein cholesterol [187]. There is also evidence for potential carcinogenetic effects of testosterone therapy on the prostate gland, although this is still controversial [188].

*Vitamin D and Calcium Supplementation*. Vitamin D deficiency is highly prevalent in COPD and, as such, supplementation may appear as a treatment option (Figure 5), particularly since such treatment was found to have beneficial effects in deficient individuals. Indeed, vitamin D supplementation has been shown to increase muscle strength in vitamin D deficient adults, to decline the odds of falling, and to reduce the risk of hip and other nonvertebral fractures in elderly [189]. When combined with calcium supplementation, it also improved balance [190], increased bone mineral density, suppressed bone remodeling [191], and improved muscle function [192, 193]. Further, the combination of vitamin D supplementation and exercise training in elderly without COPD improved gait speed, body sway, and muscle strength [194].

In patients with COPD, a few studies have examined the effects of vitamin D supplementation. A 6-week treatment with a daily dose of 2000 IU of vitamin D was found to increase vitamin D levels towards normal levels but was not associated with improved physical performance, as assessed with short physical performance battery, or with health related quality of life [195]. The supplementation of a high dose of vitamin D (100.000 IU per month) during a 3-month rehabilitation program improved maximal oxygen uptake, but not quadriceps strength or six-minute walking distance [196]. Finally, in a randomized controlled trial, although no overall reduction in exacerbations could be found after a one-year treatment with a high dose vitamin D, it was clear that, in a subgroup of patients with COPD very deficient for vitamin D at baseline, supplementation resulted in 43% reduction of exacerbations [197]. Keeping in mind the deleterious direct or indirect effects of exacerbations on skeletal muscle and osteoporosis in COPD, these data should not be neglected. 

#### 2.6.2. Nonpharmacological Interventions

*(1) Land-Based Exercise*. Pulmonary rehabilitation consisting of strength and exercise training is the most effective nonpharmacological and multidisciplinary intervention used to improve symptoms, muscle strength, and exercise capacity and health status in patients with COPD (Figure 5) [198, 199]. In patients with stable COPD, exercise training is found to improve muscle function and exercise capacity and to increase fatigue resistance [198, 200]. It is also associated with improved health status [201] and quality of life [198, 202, 203]. Interestingly, muscle strength was also found to be improved when exercise training was combined with testosterone therapy [204, 205]. In addition, exercise training improved cross-sectional area of all fiber types within the vastus lateralis muscle [206–209] and shifted quadriceps muscles fiber type distribution in favor of type I fibers [206, 207], resulting in a muscle energy metabolism shifted from glycolytic towards oxidative metabolism [210]. The morphological adaptations in peripheral muscles were similar in GOLD stages II-IV [208].

In patients with COPD hospitalized for acute exacerbations, resistance training, starting from the second day of hospitalization, was found to increase muscle force with 10% and to improve six-minute walking distance after discharge. This was associated with a more favorable anabolic/catabolic balance in the muscle. One month after discharge, functional status and muscle force were better in the group under training during exacerbation [211]. Starting pulmonary rehabilitation immediately after a COPD exacerbation was found to be highly effective and safe. It resulted in reduced hospital admissions and mortality, improved quality of life, and improved exercise capacity in patients with COPD [212].

Maintaining a physical active life is the best remedy for reducing the risk for osteoporosis and improving quality of life [149] since training is found to regulate bone maintenance and stimulate bone formation [213], including accumulation of minerals [214], and is associated with higher bone mineral density [215, 216] in healthy individuals. While there is plenty of evidence of the positive effect of physical activity on bone density and fractures in the general population, studies in patients with COPD are lacking.

*(2) Alternative Training*. For patients with COPD with severe dyspnea, older age, and physical comorbidities, water-based training is found to be an excellent alternative for land-based training. Although water-based training did not cause extra beneficial effects on walking distance, strength, and well-being in patients with COPD without any physical comorbidities [217], patients with COPD with physical comorbidities were found to be more susceptible to the beneficial effects of water-based exercises, as observed by a greater improvement of endurance exercise capacity and fatigue as well as dyspnea [218]. Water-based exercises also prevent bone loss and improve dynamic standing balance and quality of life in healthy individuals [219] as well as in older women with osteoporosis, although it did not change their fear of falling [220, 221].

Neuromuscular electrical stimulation is another intervention that was found to be very useful for severely deconditioned patients with COPD, since its load on the cardiopulmonary system is low [222], and moreover, it might be considered for home use [223]. Exercise capacity and quality of life were improved, while muscle strength was found to be increased with 20% to 30% in patients with COPD [223–226]. The cross-sectional area of the mid-thigh muscle and type II fibers was found to be enhanced, while that of type I fibers was decreased [227, 228]. A fiber shift in favor of type I muscle fibers as well as reduced muscle oxidative stress [229] was observed along with a more favorable anabolic to catabolic balance [228].

Another alternative training is the use of whole body vibration. It is a neuromuscular intervention whereby a low amplitude and high frequency (35–40 Hz) mechanical vibration is applied to the whole body through a vibrating platform. This training modality is found to improve muscle strength [230, 231], jump height [232, 233], and balance and to reduce bone fragility in healthy individuals [234]. In patients with severe COPD, whole body vibration is a promising training modality as it improves functional capacity [235], muscle force, and quality of life [236]. It is suggested that whole body vibration might even enhance the effects obtained with a conventional pulmonary rehabilitation program [236]. The optimal intensity and duration of the whole body vibration training as well as its long-term effects still need to be optimized. 

*(3) Nutritional Intervention*. Weight loss is highly prevalent in patients with COPD and this worsens during an acute exacerbation [114]. Weight loss is also known to be a negative contributor to survival in these patients [122] and it is also associated with skeletal muscle weakness [111] and osteoporosis [25]. Therefore, nutritional supplementation (Figure 5) might be a useful therapy to increase body weight in these patients. Indeed, body weight gain was found to be associated with improved prognosis of patients with COPD [122] and increased energy intake was found to improve quality of life [237].

In patients with stable COPD, the effect of nutritional supplementation is still controversial. Any caloric supplementation of more than 2 weeks did not improve weight gain, lung function, or functional exercise capacity [238, 239], while, in other studies, oral administration of high calorie/high protein diet for 3 months resulted in increased muscle strength and improved muscle contractility and fatigability [240]. Similarly, dietary counseling, food fortification, and nutritional supplementation were found to have positive effects on weight gain in undernourished patients with COPD [241, 242] but improved weight gain was predominantly due to increased fat mass. In patients with COPD who are hospitalized for an acute exacerbation, nutritional support was found to increase the caloric intake [243]. The integration of a nutritional supplementation therapy in a pulmonary rehabilitation program was found to improve body composition, muscle function, exercise capacity, and health status in undernourished patients with COPD [244].

Since many studies conclude that body mass index and fat-free mass index are related to skeletal muscle weakness and osteoporosis, adequate nutritional support is warranted, especially in patients with an already impaired energy balance. 

*(4) Fall Prevention and Balance Training*. Patients with COPD often exhibit balance problems [56] and are highly susceptible to falls [245, 246]. In healthy individuals, strengthening of the muscles during exercise programs is found to improve muscle strength and stability and to optimize bone mineral density [247] and bone strength [248, 249]. As a consequence, the risk of fall and fractures is reduced [250] and physical balance is improved [219]. Balance training as part of the pulmonary rehabilitation program also improved balance performance, muscle strength, and physical function in patients with COPD [251].

## 3. Future Perspectives

Although the literature concerning chronic obstructive pulmonary disease, skeletal muscle weakness, and osteoporosis is already very broad, after writing this review it became clear that much information is still lacking. In particular, the link between risk factors such as physical inactivity, oxidative stress, and hypogonadism and osteoporosis in patients with COPD is indirectly based on data obtained in healthy individuals. Therefore, studies revealing the impact of these risk factors on the development of osteoporosis in patients with COPD are recommended. Further, this review emphasized the importance of systematically assessing skeletal muscle function together with osteoporosis in patients with COPD, keeping in mind their high prevalence in these patients as well as their impact on quality of life and mortality. Finally, therapies and strategies that are directed at improving both comorbidities should be considered, since, for the time being, only exercise training seems to reach this goal.

## 4. Conclusion

This review emphasized evidence that skeletal muscle weakness and osteoporosis are two comorbidities of chronic obstructive pulmonary disease that most likely coexist together. These comorbidities are highly prevalent in patients with COPD; they share several risk factors, including cigarette smoke, physical inactivity, systemic and local inflammation, oxidative stress, hypogonadism, vitamin D deficiency, nutritional deficits, and age. In addition, a cluster analysis confirmed the cooccurrence of both comorbidities together in a subgroup of patients with COPD. Therefore, several therapies known to improve muscle function can be beneficial for osteoporosis as well. This should be taken into account when treating patients with COPD.
